# Circulating Cell-Free DNA in Breast Cancer: Searching for Hidden Information towards Precision Medicine

**DOI:** 10.3390/cancers13040728

**Published:** 2021-02-10

**Authors:** Maria Panagopoulou, Manel Esteller, Ekaterini Chatzaki

**Affiliations:** 1 Laboratory of Pharmacology, Department of Medicine, Democritus University of Thrace (DUTH), 68100 Alexandroupolis, Greece; achatzak@med.duth.gr; 2 Josep Carreras Leukemia Research Institute (IJC), Badalona, 08016 Barcelona, Spain; mesteller@carrerasresearch.org; 3 Centro de Investigacion Biomedica en Red Cancer (CIBERONC), 28029 Madrid, Spain; 4 Institucio Catalana de Recerca I Estudis Avancats (ICREA), 08016 Barcelona, Spain; 5 Physiological Sciences Department, School of Medicine and Health Sciences, University of Barcelona (UB), 08016 Barcelona, Spain; 6 Institute of Agri-Food and Life Sciences Agro-Health, Hellenic Mediterranean University, 71003 Crete, Greece

**Keywords:** breast cancer, biomarker, circulating cell-free DNA, liquid biopsy, prognosis, monitoring

## Abstract

**Simple Summary:**

Our research focuses in the elucidation of the nature of circulating cell-free DNA (ccfDNA) as a biological entity and its exploitation as a liquid biopsy biomaterial. Working on breast cancer, it became clear that although a promising biosource, its clinical exploitation is burdened mainly by gaps in knowledge about its biology and specific characteristics. The current review covers multiple aspects of ccfDNA in breast cancer. We cover key issues such as quantity, integrity, releasing structures, methylation specific changes, release mechanisms, biological role. Machine learning approaches for analyzing ccfDNA-generated data to produce classifiers for clinical use are also discussed.

**Abstract:**

Breast cancer (BC) is a leading cause of death between women. Mortality is significantly raised due to drug resistance and metastasis, while personalized treatment options are obstructed by the limitations of conventional biopsy follow-up. Lately, research is focusing on circulating biomarkers as minimally invasive choices for diagnosis, prognosis and treatment monitoring. Circulating cell-free DNA (ccfDNA) is a promising liquid biopsy biomaterial of great potential as it is thought to mirror the tumor’s lifespan; however, its clinical exploitation is burdened mainly by gaps in knowledge of its biology and specific characteristics. The current review aims to gather latest findings about the nature of ccfDNA and its multiple molecular and biological characteristics in breast cancer, covering basic and translational research and giving insights about its validity in a clinical setting.

## 1. Introduction

Breast cancer (BC) remains in the very top of female oncology entities, with over 2 million new cases globally in 2018 [1]. BC is a heterogeneous disease of varying progression, while drug resistance and metastasis greatly reduce the survival rates. Current diagnostic/monitoring methods include mammography, ultrasound, tru-cut biopsy and ΜRI/CT scan. These techniques have several drawbacks (e.g., not suitable for all patients, low sensitivity and specificity, invasive and expensive).

Circulating biomarkers have been gaining ground as easy, minimally invasive choices for disease follow-up. The carcinogenic antigen CA 15-3 remains the “gold standard” for disease and therapy monitoring, although inadequate in sensitivity and specificity [2,3]. The FDA has recently approved the CellSearch system for measuring circulating cancer cells (CTCs), but only in metastatic disease [4,5]. Also, Oncotype DX, a 21-gene transcript-based assay, is currently used as a prognostic tool and for personalized treatment options in early stage ER + BC [6]. As an alternative, ccfDNA is currently the spearhead in biomedical research and provides the choice of non-invasive repetitive sampling for cancer monitoring. However, still limited clinical implementation [7], while a better understanding of its biology is expected to create an opportunity for its optimal exploitation in clinical routine. In the present review, we summarize the growing evidence that support this view, focusing in data specific for BC. Besides articles referring to ccfDNA, we also include findings from studies from other circulating complexes that contribute to the ‘pool’ of the ccfDNA, such as nucleosomes, vitrosomes and extracellular vesicles. Aberrant genetic alternations detected in ccfDNA are omitted, as thoroughly recently reviewed elsewhere [8,9]. We mainly present data from human studies, in any case representing the greater majority of the relevant literature, as studies in animal models are limited, probably due to the technical problem of low abundance of biomaterial. In vitro studies contributing significantly in our understanding of the biology of ccfDNA in terms of release and role are presented separately.

## 2. Liquid Biopsy

During the life span of a tumor, cancer cells change constantly, acquiring genetic and epigenetic modifications and forming clones with different survival advantage resulting in the heterogeneity of cancer cell population [10,11]. The idea of discovering tools depicting these changes and monitoring them in «real time» is of obvious importance. Liquid biopsy is a minimally invasive approach in oncology, using peripheral blood as a source of biological material escaping the tumor and enriching circulation, such as ccfDNA, circulating tumor cells (CTCs) or extracellular vesicles (EVs) and platelets, assuming that they carry identical molecular characteristics of the parental tumor [12,13]. Liquid biopsies could reflect the heterogeneity of a primary tumor or the molecular evolution of a distant metastatic lesion, which is impossible using the conventional tissue biopsies. Another significant advantage is that upon sequential sampling due to its minimally invasive nature, it is possible to dynamically monitor disease and drug resistance acquisition. This approach could therefore offer a powerful tool in the field of clinical oncology of recognized value [14,15]. The initial steps on its actual implementation in clinical practice are taken and are expected to move forward longitudinally in the starting decade.

## 3. Circulating Cell-Free DNA

The first demonstration of circulating DNA in the bloodstream of healthy individuals was done by Mendel and Métais in 1948 [16]. Thirty years later, it was shown that the concentration of ccfDNA from cancer patients is greater than that from healthy individuals [17]. In 1989, Stroun et al. identified fragments of circulating DNA originating from cancer cells in the bloodstream, based on a technique that identified decreased strand stability [18]. These hallmarks brought circulating DNA in the center of the biomarker discovery field to aid precision medicine.

ccfDNA is DNA liberated from cells into biological fluids, e.g., blood, lymph, bile, milk, urine, saliva, mucous suspension, spinal fluid [19]. It is double or single stranded and can be either of nuclear or mitochondrial origin. In health, ccfDNA is mainly released from cells like hematopoietic, whereas in disease, it is enriched also from pathological tissues. Cancerous ccfDNA is called circulating tumor DNA (ctDNA) and represents only a fraction of the total ccfDNA in the blood [20]. ctDNA is liberated from tumor cells, metastatic sites and CTCs and it has been proved to reflect dynamically the genetic and epigenetic events in the tumor’s lifetime [21]. The detection of mutations, Loss of heterogeneity (LOH) and aberrant methylation is considered a mean of identification of the ctDNA fraction and could serve as diagnostic/prognostic/predictive indicators [14,22,23]. Minimally invasive consecutive sampling might therefore represent dynamically genetic and epigenetic characteristics of the tumor presenting a clear advantage over established biomarkers.

## 4. Methylation of ccfDNA

DNA methylation is defined as the covalent addition of a methyl group at the 5-carbon of the cytosine ring by DNA methyltransferases (DNMTs), mostly within CpG dinucleotides [24]. It is a well-defined epigenetic mechanism contributing to gene expression regulation [25]. DNA methylation is related to a variety of normal functions [24,26]. Also, promoter methylation of susceptible genes is associated with cancer [27,28,29] as well as hypomethylation [30] and their evaluation has been suggested as a potential clinical biomarker [31,32]. ccfDNA released from tumor cells has been shown to retain its epigenetic features [33,34]. Studies in multiple types of solid tumors have investigated the methylation profile of ccfDNA to evaluate its diagnostic, prognostic and predictive potential and add in their clinical management [14,22]. In breast cancer, the first documentation of aberrant methylation of ccfDNA was by Silva JM et al., in 1999, detecting the methylation of P16INK4A in plasma and in the corresponding tumor, indicating its cancer origin [35]. Since then, many studies reviewed below, have been performed to evaluate liquid methylation biomarkers in breast cancer associated with different clinical endpoints (Table 1). They differ significantly in the pre-analytical protocols for ccfDNA isolation as well as the methodology adopted for methylation detection assays and are often limited in a small cohort, still they accumulatively show that there is valuable information there awaiting further exploitation.

### 4.1. Methylation of ccfDNA as Diagnostic Biomarker in Breast Cancer

The detection of aberrant ccfDNA methylation at early carcinogenetic stages can hold diagnostic value. Many gene promoters have been found methylated more often in the plasma of BC patients in relation to healthy individuals. Firstly in 2006, it was shown that the methylation status of *RARβ2* and *RASSF1A* in ccfDNA was specific in distinguishing benign versus malignant breast tumors [36]. Since then, numerous studies have highlighted that the methylation status of other genes as detected in ccfDNA could serve for early diagnosis of BC. For instance, a gene panel of *ITIH5, DKK3* and *RASSF1A* were proposed by Kloten et al. [37]. Other potential biomarkers reported are *CST6* [38], *BRCA1, MGMT, GSTP1* [39] and a panel of *APC, FOXA1* and *RASSF1A* [40]. Moreover, Nunes et al. proposed two gene panels, the “PanCancer” (*APC, FOXA1, RASSF1A*) and “CancerType” (*SCGB3A1*, *SEPT9*, *SOX17*) for early detection of women with breast, colon and lung cancer [41]. Li et al. using next generation sequencing (NGS) found that the methylation *of EGF* and *PPM1E* genes and eight different CpG sites could lead to early diagnosis of BC [42]. A screening tool having comparable sensitivity with mammography was proposed by Uehiro and Sato. Using digital PCR technology, they suggested a multiparametric model containing 4 methylation markers, ccfDNA levels and the mean of 12 methylation markers as features for discriminating BC patients versus healthy individuals [43]. The above genes have been studied in already diagnosed BC patients, but their value as potential diagnostic biomarkers should also be examined in women of high risk in developing BC. Interestingly, in a recent prospective cohort study in which researchers studied the methylation pattern in blood samples collected from non-breast cancer women who had a sister with breast cancer, researchers found that women who eventually developed BC had methylation similar to non-cases, suggesting that methylation differences are likely a consequence rather than a cause of breast cancer [44].

The introduction of genome-wide DNA methylation approaches has led to the development of large databases enriched daily with data from whole epigenome readings from different sources (tissues, pathological entities, species etc.). Datasets archived for example in MethHC and The Cancer Genome Atlas (TCGA) provides a valuable source of information to identify potential sites of differential methylation. Moss et al. combined data from TCGA and Gene Expression Omnibus (GEO) and original data from human plasma to introduce an algorithm for tracing the tissue origin of ccfDNA in BC, using a genome-wide methylome method for early detection and therapy monitoring [45]. In addition, researchers could identify the enrichment of ccfDNA from multiple cell types and discriminate ccfDNA from different cancer types. Kang et al. using genome-wide DNA methylation data introduced the CancerLocator that could predict in ccfDNA, not only the tumor burden but also the cancer tissue origin in BC and other cancer types [46]. Recently, researchers using targeted methylation sequencing of 9223 CpG in ccfDNA could detect and classify advanced BC and other cancers with great accuracy [47]. Shen et al. introduced the cfMeDIP–seq CpGs, a technique that combined methylated ccfDNA immunoprecipitation and high-throughput sequencing for genome-wide bisulfite-free plasma DNA methylation profiling for detection and classification of early-stage cancers [48]. These recent studies point into the ground-gaining of high-throughput methodological approaches in the field of cancer diagnostics. Overall the aforementioned studies proved that tissue specificity of methylation could not only reflect tumor burden but also allow detecting specific cancer type, a great advantage for its exploitation in clinical practice.

### 4.2. Methylation of ccfDNA as Prognostic Biomarker in Breast Cancer

The methylation patterns of ccfDNA could also hold significant information related to tumor aggressiveness, the likelihood of relapse and metastasis, as well as survival. For instance, the methylation of multiple genes (*PRB, ERALPHA, RASSF1A, P16INK4A, RARBETA2, GSTP1, BRCA1*) had an important prognostic impact in BC [49]. Other genes that have been shown to have a prognostic value in BC are *CST6* [38], *SOX17* [50] and *ESR1* [51]. In a relevant study, the detection of promoter methylation of at least one from *GSTP1, RASSF1A* and *RARB2* in ccfDNA was correlated to shorter survival of BC patients [52,53]. More recently, Widschwendter et al. using bisulfite sequencing, reported that the pre-therapeutic methylation of the specific region EFC#93 in the serum of BC patients was associated with relapse within the next five years, raising its value as a prognostic biomarker [54]. In our recent work, we found that 4 genes (*KLK10, SOX17, WNT5A, MSH2*) are frequently methylated in the ccfDNA of BC patients and were correlated to prognostic parameters. Subsequently, a classification analysis by a machine learning software combined clinical data and experimental findings and produced multi-parametric prognostic signatures for metastatic BC patients, predicting survival and disease outcome [55].

### 4.3. Methylation of ccfDNA as a Biomarker Predicting Treatment Response in Breast Cancer

Among the most important applications of liquid biopsy is the monitoring of treatment response in “real time”, while the dynamic changes of ccfDNA methylation could be detected by sequential sampling. Few studies have attempted relating aberrant methylation of ccfDNA to treatment response and to drug resistance acquisition in BC, in order to unravel pharmacoepigenetic correlations [56,57]. In BC, the methylation profile of *BRCA1* was different between responders and non-responders to neo-adjuvant therapy [56]. Another study showed that *RASSFIA* methylation was disappeared upon response to adjuvant therapy, while the persistence of methylation meant resistance [58]. ccfDNA methylation of *STRATIFIN* was reported to have sufficient sensitivity and specificity to discriminate patients between disease-free and metastatic BC groups and was suggested as a marker for treatment monitoring in metastatic BC [59]. *ESR1* silencing by methylation as detected in ccfDNA was shown to affect the expression of the estrogen receptor protein in tumors of BC patients, whereas high methylation was associated with estrogen receptor negative status predicting resistance to endocrine therapy [51]. Liggett et al. reported that the pre-therapeutic levels of methylation for *PAX 5* and *RARB2* gene were decreased after surgery, whereas tamoxifen treatment changed *ESR1* methylation, suggesting their use as markers for treatment response [60]. Fackler et al. introduced the cMethDNA, a PCR methylation-based assay for the contemporary study of 10 genes for treatment response monitoring of metastatic BC, having a sensitivity of 91% and a specificity of 96% for identifying recurrent stage IV patient [61]. Legendre et al. identified 21 CpG island hypermethylated hotspots in ccfDNA of metastatic breast cancer and proposed the potential use of this signature for therapy stratification [62]. Finally, in our latest work, the increased methylation of three or four out of five genes (*KLK10, SOX17, WNT5A, MSH2, GATA3*) was associated with absence to pharmacotherapy response [55].

## 5. Hypomethylation in Breast Cancer

Breast cancer cells are highly hypomethylated [63,64,65] and global hypomethylation is correlated to clinicopathological characteristics of breast lesions [65]. A possible mechanism for DNA methylation loss in BC is through the formation of repressive chromatin at partially methylated domains (PMD) [66]. A recent study in BC reported that hypomethylation in PMD occurs in large fractions of the genome that display genetic and epigenetic alterations [67]. Only a few studies have investigated global hypomethylation of ccfDNA in BC. Genome-wide approaches have proved that ccfDNA is hypomethylated in metastatic breast cancer (MBC) [62,68]. Global hypomethylation was also detected in the plasma of BC patients by massively parallel bisulfite sequencing, which could be an attractive approach for diagnosis and disease monitoring [69].

## 6. Other Parameters of ccfDNA in Breast Cancer

The research on the development of ccfDNA-based biomarkers in cancer is not limited to the analysis of its sequence for identifying alterations (DNA methylation, mutations, LOH, etc.). Below, we present data from the study of other parameters such as quantity, protein content, integrity, release mechanism, etc. important features that could lead to the development of multi-parametric prognostic and predictive biomarkers in BC.

### 6.1. Quantity of ccfDNA

As aforementioned, small quantities of ccfDNA are detected in the plasma/serum of healthy individuals, but its concentration is notably increased in cancer or other pathological conditions [70,71]. The quantity of tumor-derived ccfDNA in the bloodstream differs and depends on tumor size and cancer type (blood-barrier in brain tumors). Also, it has been mentioned that DNAase activity often impaired in cancer patients is correlated to ccfDNA concentrations [72]. Clearance rates in liver, spleen, kidney and to a less extend degradation from blood nucleases are additional factors affecting quantity [73,74,75], while the half-life of ccfDNA could last from 15 min to a couple of hours [19].

Besides other characteristics, quantity of ccfDNA is by itself a parameter with potential value for diagnosis, classification and treatment monitoring. Several techniques have been proposed for total ccfDNA level measurements in blood, either direct in unpurified plasma [55,76,77] or after DNA isolation [78,79]. In our recent work in BC, we measured ccfDNA quantity directly, using a SYBR Green-based/Qubit assay; it is important to note that by this method, only free unbound ccfDNA is measured, as assay SYBR Green dye can only bind to free/naked DNA. In contrast, after isolation, all ccfDNA (naked, bound in nucleosomes, proteins or internalized in vesicles) is extracted and measured. The techniques mostly used so far for ccfDNA quantification is quantitative PCR (qPCR) in BC for the short and long sequences ALU115/247 [80,81] and LINE1 sequences [82] or using the reference gene *GAPDH* [83,84]. Both methods have repetitively confirmed higher ccfDNA levels in BC in relation to healthy individuals [79,81,82,84,85,86,87,88,89,90,91,92,93]. Increased levels of ccfDNA in BC have also been correlated to metastasis [55,81,86], tumor size [79,82,83,84], other histopathological parameters [79,89] and BC outcome [55,90]. In our recent study, elevated levels of ccfDNA were correlated to the incidence of death, shorter PFS and non-response to pharmacotherapy in metastatic patients [55]. Most interestingly from a clinical aspect is the construction of a single-parametric linear model using ccfDNA plasma concentration values with great discriminating power to predict response to chemotherapy [55]. However, in our patient group we did not detect correlations of quantity to clinicopathological parameters, possibly due to the different quantification methods and patient classification criteria, in concordance with some researchers [86,88]. Other studies have assessed the ccfDNA quantity in relation to diagnosis. In a study, researchers developed a qPCR assay using telomere, centromere and LINE primers and showed that the shortening of telomeric ccfDNA in plasma was correlated to BC [94]. The circulating levels of the longer fragment of ALU247 have also been shown to hold a diagnostic potential, shown to discriminate the cancer from non-cancer subjects [87]. Also, it has been shown that ccfDNA was superior to other circulating biomarkers in detecting BC. it has been found that ccfDNA as measured by qPCR for the *GAPDH* gene, was superior to serum vascular endothelial growth factor measured by ELISA in discriminating healthy from BC women [95]. A study in MBC showed that ccfDNA was superior to CTCs or CA 15-3 for disease monitoring, as levels showed greater correlation with changes in tumor burden and detected earlier than CA 15-3 or CTCs treatment response [96], proving its superiority over other innovative or established circulating biomarkers. This was further confirmed by studies using ALU and LINE1 levels to quantify ccfDNA [97,98]. It was earlier proposed that cancerous ccfDNA fragment measurements could serve as a reliable tool to monitor tumor dynamics in the course of disease and therapy [15] and indeed a recent meta-analysis of 13 studies concluded that the concentration of ccfDNA had great sensitivity and specificity [87% (95% CI, 73–94%) and 87% (95% CI, 79–93%), respectively] for BC diagnosis [99]. Furthermore, Catarino et al. using a real-time PCR probe assay for the *hTERT* gene, quantified ccfDNA of BC patients before and after surgery. They showed that ccfDNA levels were significantly decreased after surgery, successfully reflecting the tumor removal [85]. In accordance to that, Agassi et al. used a SYBR Gold-based fluorescence assay for ccfDNA quantification and confirmed that ccfDNA quantity was diminished after tumor resection [100]. Recently, researchers using the same quantification technique found that the reduction of ccfDNA levels were correlated to surgical removal or tumor reduction by chemotherapy, confirming once again previous studies. However, in the same study ccfDNA levels could not discriminate between patients with BC and healthy individuals for diagnostic purposes [101]. Maybe this discrepancy could be attributed to the use of the SYBR Gold-technique for ccfDNA quantification which can be quite sensitive, but lacks in specificity due to RNA interference. Very recently, Moss et al. compared genome wide methylation data of different tissues and cell types and found a breast-unique methylation pattern of three genes (znf296, krt19, lmx1b) which was used to quantify breast derived-ccfDNA in plasma using massive parallel sequencing. This approach could sufficiently discriminate between healthy individuals and cancer patients (AUC: 90.44% (95% CI: 78.51%–100%)), while no breast molecules were identified in healthy individuals. Also, breast derived-cfDNA levels were associated with tumor aggressiveness and a decrease was noticed during neo-adjuvant treatment. Notably, the persistent presence of breast derived-ccfDΝA after treatment indicated the existence of minimal residual disease [102]. This is an excellent proof showing that the tissue specificity of methylation could precisely reflect and monitor tumor burden. A more sophisticated approach for optimal feature selection such as automated machine learning would be a more appropriate methodological choice to deliver tissue specific signatures.

Obviously, high levels of ccfDNA in the bloodstream could be due to the presence of a solid tumor but could also be related to other pathologies such as autoimmune disorders, inflammation and others. Hence, ccfDNA concentration can be proposed to serve diagnostic proposes in BC or reflecting removal of a primary breast tumor only adjunct to other tissue of origin or cancer related markers and clinical manifestations. On the other hand, due to its high sensitivity in MBC and in predicting treatment response [55,96], it could be envisaged to offer a reliable and simple solution for treatment monitoring. Validation in a clinical setting is highly anticipated to speed up application.

### 6.2. Integrity of ccfDNA

In 1989, Stroun et al. showed that ccfDNA of cancer patients is shorter than the ccfDNA of healthy individuals [18] implying that the study of ccfDNA integrity could aid the discrimination of cancerous ccfDNA from total ccfDNA but also biomarker discovery. Many studies have been conducted analyzing ccfDNA Integrity (cfDI) as the ratio between longer and shorter DNA fragments, with controversial findings so far. The most widely used method for cfDI assessment is the measurement of non-coding DNA integrity, such as repetitive elements ALU and LINE. In a 2006 study, researchers using the ALU247/ALU115 ratio found that patients having breast cancer of stage I, II and III showed greater integrity of ccfDNA as compared to healthy individuals [89]. Similarly, Iqbal et al. showed that ALU247/ALU115 was higher in stage IV breast cancer than in earlier stages and declined after surgery, suggesting it as a clinically relevant prognostic biomarker [90]. Kamel et al. found that cfDI was significantly higher in breast cancer than in benign breast patients and healthy individuals, using different amplicons of β-actin and was correlated to TNM stage [103]. Similar studies have been conducted in breast cancer and other cancer types confirming the finding that cfDI is greater in cancer [89,93,104,105].

These results however were not confirmed by several other studies, showing in contrast that healthy individuals showed greater cfDI than BC patients. Madhavan et al. suggested that it is the reduced cfDI that can serve as diagnostic marker for primary and metastatic breast cancer [106]. In a later study, researchers using the long and short fragment of *HER2, MYC, BCAS1* and *PI3KCA* genes showed that BC patients had lower integrity than healthy individuals [91]. Cheng et al. using the ALU/LINE1 method proved that in BC the cfDI was significantly lower in recurrent patients, discriminating them from the non-recurrent patients [107]. Also, the same researchers reported that MBC patients showed increased cfDI after the first cycle of therapy and that it can be an independent prognostic marker [97] in contrast to earlier findings showing that the distribution of the cfDI in BC patients did not change after adjuvant chemotherapy [108]. Both Cheng’s and Madhavan’s studies used greater BC cohorts [106,107] than previous studies [89], adding to the power of their findings, however this controversial matter needs further elucidation.

Massive parallel sequencing added considerable to the deeper understanding of ccfDNA integrity. Jiang et al. proved that fragments originating from cancer cells were smaller than the fragments from healthy cells in patients with hepatocellular carcinoma. In the same study, patients having greater quantity of cancerous ccfDNA had a more fragmented DNA profile [109], in concordance with two previous studies in metastatic colorectal cancer [110] and pancreatic cancer [111]. In our BC study, we showed via capillary electrophoresis that patients at advanced stage that started neo-adjuvant or first line therapy had fragments sized from 22 to 160 bp, whereas this pattern was not observed in healthy individuals. We also showed that patients with higher total levels of ccfDNA had a greater number of short fragments (<160 bp). Finally, tumor size and the incidence of death were correlated with greater DNA fragmentation [55]. We assume that the pattern of fragments (22 to 160 bp) that we found in advanced BC is the result of degradation after ccfDNA liberation during cell death or active release. Most recently, researchers used a genome-wide approach for analyzing the fragmentation patterns of ccfDNA for early detection of BC and six different cancer types (DELFI study). They found that healthy individual ccfDNA patterns were correlated to nucleosomal DNA fragments originating from lymphocytes, while cancer patient fragmentation patterns were more variable, with shorter median overall length, in concordance with our findings. Most interestingly, using the DELFI approach they could recognize with high sensitivity a specific cancer type among others [112].

cFDI assessment could have a clinical application, although there is still discrepancy between researchers, some claiming that longer fragments represent the tumorous DNA while others the opposite. We assume that the main reason for these controversial findings is the selection of different methods for measuring cfDI (ALU247/ALU115 vs. others), as different fragments are quantified. Other reasons possibly depend on the differences in studied patient groups, with varying disease stage representation. Tumor growth kinetics may cause significant differences in the cellular release of ccfDNA and degradation. The pre-analytical process chosen in each study might as well represent a source of discrepancy, as shown in a study comparing different extraction methods of ccfDNA from plasma (phenol-chloroform isoamyl vs. QIAamp DNA Blood Mini Kit) that found different fragment lengths in the elutant of each method [77]. Despite the fact that ccfDNA is systematically investigated, until now different groups have not agreed to a standard operational pre-analytical procedure (e.g., sample collection, DNA extraction method), leading to variations and often in opposite findings between studies. In conclusion, for valid conclusions drawn from ccfDNA integrity studies, but also in general, it is important for the different methods to be compared in the same cohort of samples, as well as the establishment of a widely-accepted pre-analytical procedure.

### 6.3. ccfDNA Releasing Mechanism

Both apoptosis [113] and necrosis [71] have been suggested as mechanisms of cellular release of ccfDNA, whereas active release from viable cells [114] has also been described. Different ways of cell death are also sources of ccfDNA. For example, macrophages which engulf and degradate necrotic and apoptotic cells liberate degraded DNA [115]. An ischemic cell death (oncosis) has also been described in cancer [116] and could alternatively release DNA fragments. ccfDNA of 166 bp or multiples (single, di-, tri- and polynucleosomes) is possibly released through apoptosis and is the result of the action of a caspase-dependent endonuclease that cleaves DNA between nucleosomes. It is more or less accepted that the larger fraction of ccfDNA in human plasma is produced via apoptosis [ 109], fragments sized 10,000 or bigger derive from necrosis, while active release delivers a fragments of 2000 bp [117,118], although it is clear that the exact pathways of ccfDNA production in each case still needs to be clarified. Our study evaluated fragment size distribution by capillary electrophoresis and showed all above types of fragments (160 bp, 2000 bp and 10,000 bp) present in the plasma of BC patients, indicating all three releasing mechanisms (apoptosis, active release and necrosis) responsible for the liberation of ccfDNA [55]. This was further confirmed by our in vitro studies using the human breast cancer cell line MCF-7, where fragment—size profiling was indicative of active release, whereas exposure to the demethylating agent 5—AZA—CR induced the release of additional shorter fragments, indicative of apoptosis (see below) [34].

#### 6.3.1. Circulating Structures of ccfDNA

**Figure 1:**
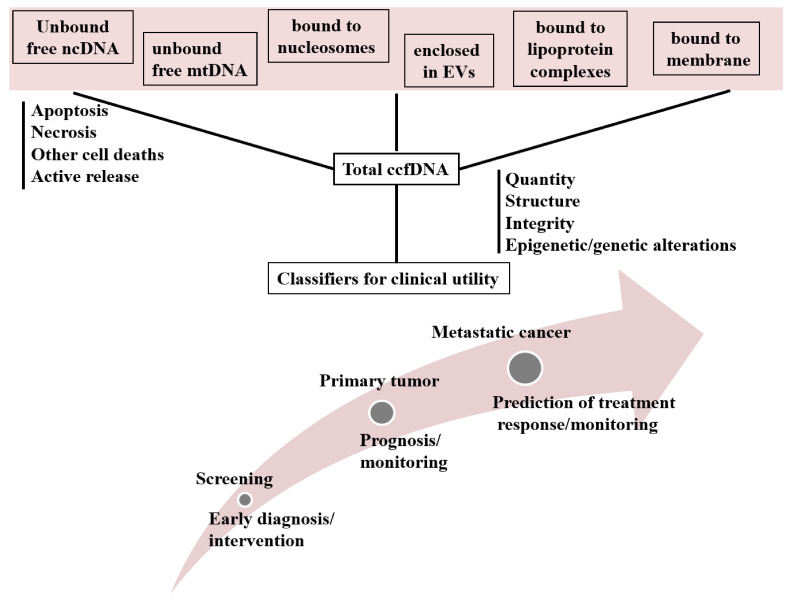
Circulating structures of ccfDNA in human plasma and their applications in clinical management of cancer. Abbreviations: BC = Breast Cancer; ccfDNA = circulating cell free DNA; Evs = Extracellular Vesicles; MBC = Metastatic Breast Cancer; mtDNA = Mitochondrial DNA; ncDNA = Nuclear DNA.

ccfDNA consists in different forms and this clearly depends on its cellular origin (Figure 1).

Nuclear originating ccfDNA is liberated in the bloodstream either as free DNA (unbound DNA) or bound to protein or lipoprotein complexes (nucleosomes, vitrosomes, fragments of cellular membranes) [119,120,121] or enclosed in EVs such as exosomes, apoptotic bodies and microvesicles (MVs) [122,123]. DNA that is enclosed in exosomes is called exosomal DNA (exoDNA), while apoptotic bodies usually contain nucleosomes, protecting them from DNAses and RNAses [124,125,126]. ccfDNA of mitochondrial origin (cf mtDNA) also circulates in the bloodstream, either free or bound to fragments of mitochondrial membranes [127]. Generally, researchers describe EVs carrying DNA, ccfDNA or nucleosomes as different circulating entities, but in translational research, ccfDNA after the isolation procedure from plasma or serum is originating from all the above structures, giving the total ccfDNA for downstream analysis and biomarker discovery. Therefore, in the present work we consider that all the above structures contribute different forms of ccfDNA. Below, we review knowledge regarding these structures in cancer, highlighting data relevant to BC management.

#### 6.3.2. Nucleosomes

Τhe basic repeated structural unit of chromatin is the nucleosome. It contains a core of a complex of histone otcamer (H2A, H2B, H3 and H4) and DNA 147bp long wrapped around it. DNA that is bound to nucleosomes is protected from degradation and nucleosomes are circulating as mono or oligo-nucleosome fragments [128] giving a specific DNA pattern (166 or multiples). Often, nucleosomes are enclosed in apoptotic bodies and engulfed from macrophages [124] and they have been shown to be able to cross the cellular membrane [125].

Several studies have been conducted in order to elucidate the value of nucleosomes as circulating biomarkers, mostly assessed by ELISA-based techniques. As for breast cancer, researchers showed that patients having benign or malignant tumor had higher Circulating Nucleosomes Levels (CNLs) than healthy individuals and levels were correlated to the presence of metastasis [129]. Many studies have proven that low CNLs were significantly associated with response to treatment in various malignancies and their quantification has been proposed for guiding treatment in cervical and in non-small cell lung cancers [130,131]. Holdenrieder et al. suggested that circulating nucleosomes could be a useful biomarker for treatment monitoring in BC between other types of cancers [132]. In addition, a study concluded that CNLs could predict neoadjuvant treatment response in locally confirmed BC [133]. Prognostic value of CNLs has also been proposed. Kuroi et al. using ELISA showed that BC patients with high CNLs had higher survival rate, although no correlation to clinicopathological features was observed [134].

In a more recent study, researchers showed that specific nucleosome footprints could reveal certain cell types, giving insights into their tissue origin [119]. Also, the detection of disease-associated epigenetic profiles of nucleosomes via a method based on ELISA could sufficiently discriminate pancreatic and colorectal cancer from healthy individuals [135,136]. Tamkovich et al. showed via MALDI-TOF Mass Spectrometry that circulating nucleosome complexes in BC patients contain tumor-associated proteins and provided further information for nucleosome bound ccfDNA [137].

In parallel to nucleosome quantification, nucleosome histone modifications such as methylation and acetylation have been correlated to prognosis, phenotype [138], diagnosis [139] and treatment response [140] in BC. It will be very interesting to monitor these modifications in plasma/serum to unravel tumor or tissue-specific information. To our knowledge, only one study addressed this issue and showed that SAT2 levels on H3K9me3 and H4K20me3 are upregulated in BC patients’ serum, while control and patients’ group were better discriminated when these values were normalized to the total nucleosomes levels [141]. More studies measuring histone modifications in plasma are needed in BC in order to prove any prognostic/predictive relevance of these markers. Also, sequential sampling and measuring together nucleosomes and histone modifications in important clinical endpoints during the course of therapy in breast cancer would be of great importance for proving circulating nucleosomes clinical value and for personalized treatment options.

#### 6.3.3. Vitrosomes

Other molecular lipoprotein-nucleic acid complexes identified in the bloodstream are the vitrosomes, which carry DNA too and protect nucleic acids from degradation. They also have been reported to act as intracellular messengers [114,121]. Vitrosomes are liberated by active release from viable cells [142]. It has been showed that they can be received from cells, chance their phenotype through oncogenic transformation and possibly lead to the initiation of metastasis [121,143]. Till now, there are no available studies on the possible role of vitrosomes in BC. Vitrosomes carrying DNA might be a valuable marker for cancer and therapy monitoring and its role in ongogenic transformation and metastatic cascade in BC should be investigated.

#### 6.3.4. Extracellular Vesicles 

The term EVs refers to the membrane vesicles found in the extracellular environment. They have been characterized and categorized based on their size, content, biogenesis and release mechanism. They seem to hold a role as intracellular messengers [144,145]. EVs carry DNA, mtDNA, mRNA, non-coding RNA, proteins and lipids, protecting their cargo from degradation and probably transferring it from a parental to a recipient cell [146,147,148]. The main vesicles circulating in human blood are exosomes, microvesicles (MVs), ectosomes and apoptotic bodies [149,150].

It has been demonstrated that EVs participate in the carcinogenic process and metastasis initiation, having a role in intracellular communication and transfection of healthy cells [151,152]. Tumor derived vesicles also contain single-strand DNA reflecting the genetic status of tumor cells [153], holding some value as a potent source of liquid biopsy. EVs have also been correlated to drug resistance. In a recent study, Keklikoglou et al. showed that cytotoxic chemotherapy could elicit the release of pro-metastatic EVs enriched with pro-metastatic molecules ANXA6 and Ly6C+CCR2+ in mouse BC models. Also, they found increased levels of ANXA6 in plasma EVs from BC patients undergoing neo-adjuvant treatment, that declined at the end of therapy reflecting treatment response [154].

A certain type of EVs, the oncosomes (100–1000 nm), named after their cargo, contain molecules of cancer metabolism and are enriched in oncogenes that could be horizontally transferred from a parental to a recipient cell [155]. In a recent study, researchers showed that the ccfDNA in the plasma of prostate cancer patients is mainly enclosed in oncosomes and carries molecular alterations identical of its cell of origin [156]. These data support our view to consider naked free DNA (unbound DNA) and DNA bound to protein or lipoprotein complexes as a whole.

Apoptotic bodies are the largest type of extracellular vesicles carrying nuclear fragments and organelles such as mitochondria. Apoptotic bodies originating from cancer cells are enriched with tumor DNA and can be horizontally transferred via uptake from recipient cells [157]. Formations of giant vesicles (3–42 μm), having aqueous content, have also been reported in breast cancer cell lines under the stimulation of 17-beta-estradiol and are also identified in human BC tissue and in murine models [158], but their contribution in the ccfDNA of the plasma has not yet been assessed.

Based on their size, another distinct type of EVs are the microvesicles (100–1000 nm) that are actively released from the plasma membrane [159]. Tumor-derived microvesicles (TDMs) contain DNA reflecting the genetic status their cell origin, although their contribution to the ccfDNA has also not been assessed. They also carry retrotransposon RNA transcripts that can be transferred in recipient cells [153]. Like other EVs, TDMs in BC contain pre-invasive molecules like extracellular matrix metalloproteinase inducer (EMMPRIN) that contribute to the tumor invasion in the surrounding tissue [160]. The number of TDMs found in the plasma of BC patients was correlated to disease stage [161] implying a value.

Exosomes, the better-studied form of EVs, are small membrane vesicles (30–100 nm) of endocytic origin [145,162]. Exosomes are secreted by almost all cell types and can be horizontally transferred to recipient cells [163]. In a 2014 study, researchers showed for the first time that double strand DNA present in exosomes represents the whole genomic DNA, while cancer derived exosomes bear the mutational status of parental cells, illustrating their potential as biomarker in clinical settings [164]. When exosomes are received by non-malignant cells, they contribute to horizontal cellular malignant transformation [165]. Melo et al. showed that exosomes from BC patient cells and serum lead non-tumorigenic epithelial cells to form tumors in a Dicer-dependent manner [166]. However, there is evidence to indicate that their DNA content is at least not the only factor responsible for this activity. Tumor derived exosomes in breast cancer are also enriched with cancer associated proteins [167,168] and miRNA [166] that could hold diagnostic, prognostic and therapy monitoring information. Exosomal miRNAs have also been correlated to cancer aggressiveness [169], angiogenesis [170], metastasis [171,172,173] and drug resistance [174] in BC. It has been proposed that exosomes are responsible for acquired resistance in an in vitro study showing that exosomes transfer drug resistance to recipient cells via P-glycoprotein (P-gp) [175]. Another study showed that HER2-overexpressing cells released exosomes carrying HER2 molecules that inhibited Trastuzumab activity [176].

Overall, it is clear from experimental efforts of over a decade that tumor derived EVs found in distinct forms based on size and content, carry the molecular footprint of parental cells and that are participating in the carcinogenic process, including metastasis and drug resistance. They also contribute with their DNA cargo to the ccfDNA assessed as a liquid biopsy biomaterial. More studies are needed to elucidate their role and most importantly their clinical validity in BC.

#### 6.3.5. Circulating Cell-Free Mitochondrial DNA (cf mtDNA)

ccfDNA is mainly considered to be of genomic DNA origin. However, it is clear that mitochondria also contribute their own circular genome in the circulation. cf mtDNA consist of shorter DNA fragments differential to the nuclear ccfDNA [109]. It has been reported that cf mtDNA exist in circulation in low abundance due to the higher susceptibility to degradation lacking histone protection [109].

Mutations copy number variations and other alterations have been described in mtDNA in cancer tissues [177,178,179,180]. In BC however, limited sensitivity has been demonstrated in tracing tumor-specific and somatic mutations in cf mtDNA [181,182,183]. On the contrary, researchers pointed a prognostic potential of tumor-derived mutant cf mtDNA in oral cancer [184].

In terms of quantity, higher levels of mtDNA have been found in cancer than healthy tissues, but mtDNA was decreased under cancer progression [185,186] and in BC cell lines and tissues, low content of mtDNA was associated to worse prognosis [187]. On the other hand, results measuring levels of cf mtDNA are conflicting. In an early study, levels have been found to be significantly lower in BC than in healthy individuals and cf mtDNA could distinguish between BC cases and healthy individuals [84]. The opposite findings were demonstrated in other studies, as levels of cf mtDNA were higher in different cancer types [188,189,190,191], including BC [192,193]. Higher levels of cf mtDNA were also correlated to unfavorable clinicopathological characteristics in BC [192,194] and BC risk [193]. It is postulated that cf mtDNA might have a diagnostic value in BC in terms of quantity upon standardized pre-analytical and assaying procedures, but methods of greater sensitivity and accuracy are necessary for reliable analysis in tracing tumor specific mutations and other alterations.

## 7. ccfDNA Biology in BC: Evidence from In Vitro Studies

Despite the growing interest in studying ccfDNA-based potent clinical biomarkers, little is known about its biological role in cancer. Several studies described above have assessed the involvement of EV carrying cancerous ccfDNA in carcinogenesis, metastasis and drug resistance, although it is not clear if it is their nucleic acid or their protein content or both responsible for the observed effects. A few in vitro studies have attempted to study ccfDNA biology in BC, thus avoiding in vivo confounding factors. It has been shown that ccfDNA is released in cell culture medium and it can be quantified in cell supernatants [114,195]. In vitro studies also have shown that the main cellular releasing mechanism of ccfDNA in breast and other cancer cell cultures is active release [34,122,196,197]. In BC cell lines, active ccfDNA liberation partially occurred via exosomes [122], but more studies are needed to unravel circulating forms of ccfDNA in vitro as well as their effect in recipient cells. It has been shown that ccfDNA is recognized by the Toll-Iike Receptor 9 (TLR9) and contributes to cancer progression [122,198]. Hence, ccfDNA could stimulate the proliferation of HR+ breast cancer cells by activating the pathway TLR9-nuclear factor kappa B-cyclin D1 pathway [122]. Furthermore, DNA from chemotherapy-killed cancer cells could transfect living cancer cells and mediate invasion via TLR9, whereas TLR9 affected response to pharmacotherapy by a TLR9-mediated inflammation, as shown in a mice BC model bearing tumors overexpressing TLR9 [198]. In our recent in vitro work, we showed that ccfDNA was liberated from human breast and cervical cancer cell lines, MCF7 and HeLa respectively, carrying identical gene promoter methylation patterns to those of parental cells. Moreover, we showed that the main mechanism of the in vitro ccfDNA liberation was active release, whereas treatment of cells with the demethylating agent 5-Azacytidine induced increase of ccfDNA via active release and apoptosis [34]. The above studies showed that cell cultures are suitable models for studying ccfDNA biology in cancer.

## 8. Multi-Parametric Analysis of ccfDNA Features Using Machine Learning Approaches

It has become widely accepted in the biomarker discovery scientific community that a single biomarker is unlikely to bear the performance characteristics it terms of sensitivity and specificity to reliably reflect cancer profile, heterogeneity and pharmacotherapy response. Scientists try to measure single ccfDNA parameters (e.g., quantity, integrity, sequence alterations, structure, cellular origin and others) against the noisy non-tumoral background of clinical samples and often fail to reveal statistically relevant associations that can be translated into outcome predictions upon clinical validation. This is probably why very few tests based—between others—on ccfDNA have made it to mature clinical development. In parallel to the massive progression of the machine learning and deep learning methodology invading biomedical science, and the availability of simplified tools requiring minimum bioinformatics expertise, a multi-parametric approach is becoming increasingly promising. This technology allows complex implementation of combining different liquid biopsy measurements and even clinical and demographical data to produce classifiers with improved strength than the weak signal of each feature and to construct signatures of high accuracy. Machine Learning (ML) uses artificial intelligence that trains systems to automatically learn and improve from experience without human interaction. ML automatically builds a model from available data for a given task to describe a relationship. The greater the quality and size of the input data, the better the performance of a model [199,200]. ML combines the minimum number of features (biomarkers, clinical, demographical data) to achieve the best performing result, creating specific signatures that could reflect dynamically the cancer state and predict outcome. In our previous work, we used for the first time an innovative, fully automated, machine learning pipeline [Just Add Data v0.6 tool (JAD Bio; Gnosis Data Analysis; www.gnosisda.gr)] [200] for predictive analysis, combining our liquid biopsy-based experimental parameters against clinically important endpoints to create a multivariable predictive/diagnostic model and identify the minimal-size set of biomarkers that collectively and optimally classify the outcome. A total of four classifiers of great performance were produced (AUC ranging from 0.737 to 0.803), selecting and integrating features of BC ccfDNA and clinical data [55]. Their further optimization and clinical evaluation in prospective designs are currently scheduled. To our knowledge a few more studies have attempted to use machine learning approaches based on liquid biopsy parameters in BC. In a relevant study, researchers used a machine learning based algorithm for the early detection of eight cancers (including BC). The Cancerseek test combined protein and mutation measurements and could detect the underling cancer type with a sensitivity ranging from 69 to 98% [201]. The DELFI study evaluated DNA fragment patterns in ccfDNA by genome-wide analysis and using machine-learning techniques it succeeded to discriminate with high sensitivity specific cancer types including BC. Further, upon combination with mutational analysis of ccfDNA the sensitivity was increased [112]. Shen et al. also introduced machine-learning for evaluation of the performance of cfMEDIP data in tumor classification [48]. ML has also been used for tumor ccfDNA fragment size analysis, for discriminating cancer patients from healthy individuals [202]. Apparently, ML could aid a lot in the development of circulating biomarkers and raise the performance of liquid biopsy-based tests and it is anticipated to be included in the standard pipeline of the development process.

## 9. Conclusions

Liquid biopsy has gained much attention as an easy and a minimally invasive procedure, enabling the request of blood-based biomarkers for cancer monitoring and personalized treatment options. ccfDNA is considered a valuable biosource for tracing molecular characteristics of a tumor, most importantly its methylation profile could imprint the tissue origin of cancer and sufficiently reflect tumor burden. Despite the above facts, there is not an available test in clinical practice for BC. A major technical problem hampering the introduction of ccfDNA in clinical practice is the lack of a standard operational procedure for its pre-analytical preparation, leading to variations in ccfDNA measured parameters between studies. Furthermore, adoption of different ccfDNA quantification methods results in a lack of standard level ranges and discrepant results arise when compared to clinical data and endpoints. Hence, standardization of a common operating procedure between studies is highly recommended.

Proof-of-principal studies have shown that DNA methylation of multiple genes in ccfDNA could lead to diagnostic, prognostic and predictive biomarkers in BC. The opportunity for sequential sampling in clinically relevant hallmarks is among the greatest advantages of liquid biopsy for “real-time” dynamic monitoring of changes in the tumor epi/genetic profile in the course of disease progression and therapy. Furthermore, the introduction of high-throughput techniques for whole epigenome reading has added a considerable advantage in the research of liquid biopsy. On the other hand, the best studied parameter of ccfDNA in BC is quantity. It is well-documented that ccfDNA is greater in BC patients than in healthy individuals and it has been correlated to diagnosis, prognosis, prediction and clinocopathological features in multiple studies, although combination with other parameters seems to be needed for greater sensitivity. This is not the case in MBC, where ccfDNA quantity (possibly related to the presence of a tumor mass in the body liberating DNA quantities) could be developed as an independent biomarker for treatment monitoring, as it has been shown to have adequate performance characteristics. ccfDNA fragmentation pattern is still under debate, as studies in BC are conflicting, requiring further studies comparing methods in the same cohort to draw conclusions. In addition, the study of different circulating forms of ccfDNA could add considerably to the faster introduction of ccfDNA in clinical practice. Higher CLNs have been measured in BC and have been suggested for treatment monitoring. Also, the study of the epigenetic profile of ccfDNA which is bounded to nucleosomes would be very informative in BC. It has been proved that tumor derived EVs carry the molecular footprint of parental cells and that their nucleic acids participate in the malignant transformation and the initiation of metastasis. More studies are needed to elucidate their effect in cancer progression, but more importantly, their clinical validity in BC. Finally, higher levels of cf mtDNA have been correlated to BC and might have a clinical value, but more advanced methods are necessary for reliable analysis of sequence alterations in cf mtDNA.

**Figure 2:**
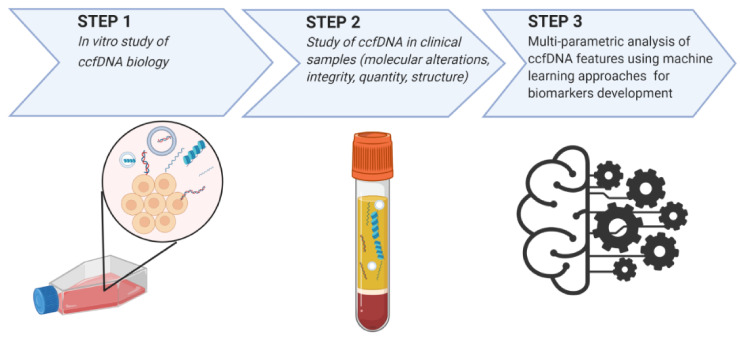
Research and development of ccfDNA-based biomarkers for the management of breast cancer. Abbreviations: ccfDNA = circulating cell free DNA.

In the era of machine and deep learning, it is becoming widely recognized that a multi-parametric approach on many ccfDNA-based features (e.g., methylation, quantity, integrity, structure) is more sufficient and could strengthen sensitivity and specificity (Figure 2). Lately ML techniques for analyzing emerging biomarkers have gained remarkable attention. ML pipelines combine the minimum number of studied biomarkers to achieve the best result. Thus, ML creates specific algorithms/classifiers that could reflect dynamically the cancer state and predict outcome, implementing higher experimental data extrapolation and accelerating the development process and their introduction into the clinical practice. Validation and optimization of suggested such classifiers in a clinical setting is promptly anticipated and expected to change the scenery in BC management.

## Figures and Tables

**Table 1:** Summary of studies evaluating ccfDNA methylation in BC diagnosis, prognosis and treatment response.

Table	Study Group	Clinical End-Point	Findings	References
Diagnostic Biomarkers				
P16INK4A	- 35 BC patients		Tumor-related origin of ccfDNA	[35]
HIC-1, RARβ2, RASSF1A	- 20 BC women - 15 women with fibroadenoma - 10 healthy women	BC diagnosis	RARβ2 and RASSF1A methylation in combination with ccfDNA quantitative analysis could discriminate malignant from non-malignant disease.	[36]
gene panel	- 250 BC women - 237 cancer-free women - 59 women with benign breast disease - 58 colon cancer women	BC diagnosis	ITIH5, DKK3, and RASSF1A methylation was correlated to early diagnosis	[37]
CST6	- 27 women with operable BC - 46 women with MBC - 37 healthy women - an independent cohort of 123 women with operable BC	clinicopathological parameters and outcome	CST6 is highly methylated in BC ccfDNA and could serve as biomarker	[38]
BRCA1, MGMT, GSTP1	- 100 BC women - 30 healthy women	clinicopathological parameters and prognosis (DFS, OS)	Concordance between tumor and ccfDNA methylation of BRCA1, MGMT, GSTP1, correlation between MGMT protein loss and promoter hypermethylation, prognostic value of BRCA1,GSTP1 methylation in ccfDNA	[39]
Gene panel	- 44 BC patients - 39 healthy individuals	clinicopathological parameters and outcome(DFS, DSS), diagnosis	Diagnostic value of APC, FOXA1 and RASSF1A methylation of ccfDNA in BC (over 70% sensitivity, specificity)	[40]
Gene panel	- 108 BC women - 72 CC women - 73 LC women	Diagnosis of BC, CC and LC, correlation to clinical parametes	«PanCancer» panel (APC, FOXA1, RASSF1A) for detecting cancer (72% sensitivity and 74% specificity) and «CancerType» panel (SCGB3A1, SEPT9, SOX17) indicating cancer topography (over 80% specificity), RASSF1A and RARβ2 methylation correlated to clinical parameters in BC	[41]
Gene panel	- 86 BC patients - 67 healthy women	BC diagnosis	EGFR, PPM1E and 8 gene-specific CpG sites were significantly hypermethylated in BC with sufficient performance for breast cancer detection (AUC 0.66 TO 0.75)	[42]
Methylation array	- Public methylation data (32 normal people, 5 BC women and patients with other cancer types)	BC diagnosis	CancerLocator tool for determining presence and location of BC	[46]
9223 CpG sites	- 15 BC - 22 NSCLC - 12 Melanoma - 29 CC patients	BC diagnosis, prognosis (OS), response to treatment (TTF)	Methylation scores could detect BC and classify the underlying cancer type with high accuracy (91.7% and 72.7% respectively), low methylation scores were associated with longer OS	[47]
Prognostic Biomarkers				
Gene panel	- 101 BC women	Prognosis (OS, DFS), correlation to clinicopathological parameters	High methylation of seven genes was correlated to poor prognosis, Methylation of p16INK4A, BRCA1, GSTP1, PRB and RARβ2 were associated with unfavorable clinical parameters	[49]
ESR1	- 110 BC women	Correlation to clinicopathological parameters	High methylation of ESR1 was associated with ER negative receptor status and phenotypes with poor prognosis and could predict treatment response.	[51]
GSTP1, RASSF1A, RARβ2	- 336 ΒC women	Correlation to clinicopathological parameters, prognosis (OS, DFS)	Positive methylation of at least one of the three genes and high ccfDNA levels were associated with worse DFS and OS	[53]
GSTP1, RASSF1A, RARβ2	- 120 BC women	Correlation to clinicopathological parameters, prognosis (OS, DFS) and response to treatment	Positive methylation of at least one of the three genes and high ccfDNA levels were associated with worse DFS and OS and no response to treatment	[52]
Six BC specific DNAme patterns	- 460 women who developed BC within three years after serum donation - 465 women who did not develop cancer the following five years	Prognosis (OS, DFS) and response to treatment	EFC#93 serum DNAme positivity was a poor prognostic factor and correlated to response to anti-hormonal treatment	[54]
Gene panel	- 200 BC women - 35 healthy women	BC diagnosis, prognosis (OS, DFS) and treatment response	Methylation of SOX17, WNT5A, KLK10 was correlated to poor prognosis and two specific classifiers were constructed for prognosis of patients with metastatic BC (AUC 0.737). Another classifier could sufficiently discriminate BC disease (AUC 0.844). Positive methylation of at least 4 of any studied gene was correlated to the absence of chemotherapy response	[55]
Predictive Biomarkers				
Gene panel	- 40 BC women (six sequential sera samples from each) - 30 healthy women	Neoadjuvant treatment response, correlation to clinicopathological parameters	*BRCA1* methylation status discriminate responders from non-responders	[56]
*RASSF1A*	- 148 BC patients (pretherapeutic and one-year-after surgery sampling)	Correlation to clinicopathological parameters, monitoring of adjuvant tamoxifen therapy response, prognosis (OS, DFS)	Methylation of *RASSFIA* was correlated to poor prognosis and resistance in tamoxifen treatment	[58]
*ESR1, STRATIFIN*	- 111 BC patients	Development of metastasis, response to treatment	Methylation of *STRATIFIN* could discriminate metastatic BC patients form those who were cancer free and was associated to treatment response (75% sensitivity and 66.7% specificity)	[59]
Gene panel	- 20 BC patients (sequential sampling)	Treatment monitoring	Methylation of *PR, PROX, MDGI, PAX 5* and *RARβ2* was diminished after surgery, especially in the combined treatment group (surgery and tamoxifen treatment). Surgery alone decreased methylation in *PAX5* and *RARβ2*, while tamoxifen treatment changed *ESR1* methylation	[60]
Gene panel (cMethDNA)	- 28 healthy, 24 BC women (training set) - 27 healthy, 33 BC (Test set)	Treatment response	Cancer-specific methylated DNA was detected in recurrent stage ΙV BC patients (91% sensitivity and 96%specificity) and cMethDNA assay could reflect treatment response	[61]
Whole-genome bisulfite sequencing	- 80 BC patients - 40 healthy individuals	Prediction of recurrence	Identification of 21 DNA hypermethylation hotspots associated with metastatic BC.	[62]

BC = Breast Cancer; CC = Colon Cancer; ccfDNA = circulating cell-free DNA; DFS = Disease Free Survival; DSS = Disease Specific Survival; LC = Lung Cancer; MBC = Metastatic Breast Cancer; NSCLC = Non-Small Cell Lung-Cancer; OS = Overall Survival; TTF = Time to Treatment Failure.

## Abbreviations

BC	Breast Cancer
ccfDNA	circulating cell free DNA
Evs	Extracellular Vesicles
MBC	Metastatic Breast Cancer
mtDNA	Mitochondrial DNA
ncDNA	Nuclear DNA
